# Supplementary material for: Distinct neurocomputational mechanisms support informational and socially normative conformity
Source: PLoS Biol. 2022 Mar 3;20(3):e3001565. doi: 10.1371/journal.pbio.3001565 (PMC8893340; doi:10.1371/journal.pbio.3001565)
Supplement: S1 Text — (DOCX) [file pbio.3001565.s001.docx]

**S1 Text**

**Alternative model for Figure 4 analysis in the main text:**

For the equivalent fixed-effect model in equation (2) in the main text (where all variables were fixed effect but not random effect) we found a strong negative correlation between the beta weights of influence and the beta weights of the interaction term (Pearson r = -.88, p<.001). This suggests that the weight for influence and interaction are constant across subjects. We, therefore, applied the following modified model:

$revision=\beta_{0}+\beta_{1}\times c_{t}+ \beta_{2}\times{inf}_{t-1}\times\left( 1+\lambda\times c_{t} \right)$ (supplementary equation 1)

where $\lambda$ is a subject independent parameter while $\beta_{0}$- $\beta_{2}$ can vary across subject. Using a mixed model approach supplementary equation 1 was fitted to the behavioural data for a fixed value of lambda. Lambda was varied on a 1000 point equidistant grid between -2 and 0. This procedure yielded a maximum likelihood estimation of $\lambda$ = -.19.

We therefore also modelled dACC BOLD using this model (supplementary equation 1, replacing revision with dACC BOLD) with the optimised $\lambda$ = -.19.

Consistent with the previous model, in the human condition, confidence had a positive effect on dACC BOLD (Wilcoxon sign-rank test W = 170 P = .01, Figure S1). The effect of influence that was modulated by confidence ($\beta_{2}$ in supplementary equation 1) did not reach significance in this model (Wilcoxon sign-rank test W = 141 P = .09, Figure S1). The absence of an influence effect might be due to greater variability in using normative information among participants. We therefore predicted that the neural effect of influence might be greater in participants who showed a behavioural effect of influence on revision. This means that we expect a correlation between the behavioural effect of influence on revision ($\beta_{2}$ in supplementary equation 1) and the effect of the same regressor on BOLD across participants. To test this hypothesis, we correlated behavioural and neural betas of this regressor across participants. Interestingly, we found that participants with higher behavioural effect of influence on revision, had also greater effect of influence on BOLD (Pearson r = .45, p = .04, Figure S2). Note that, to compute this correlation we controlled for the behavioural and neural betas of confidence using partial correlation. It should be noted that the correlation changes after removing the outlier (Pearson r = .34, p = .18). When we repeated this analysis for the computer partner condition, we found a positive effect of confidence as before, and we did not find an effect of influence on dACC BOLD.

We conclude that both models (modified model and the one used in the main text) lead to the same finding: that confidence and influence have a positive effect on BOLD in dACC, but the effect of influence is negatively modulated by the size of confidence and is restricted to the Human partner condition.

Figure S1: the effect of confidence (red) and influence modulated by confidence using the modified model (blue) on BOLD is depicted. Data and codes to recreate the figure are available at <https://github.com/alimahmoodia/Reciprocity_Data/tree/main>.

Figure S2: The behavioural (x-axis) and neural (y-axis) effect of influence on dACC BOLD ($\beta_{2}$ in supplementary equation 1) is depicted for each participant. Data and codes to recreate the figure are available at <https://github.com/alimahmoodia/Reciprocity_Data/tree/main>
