# Supplementary material for: Distinct neurocomputational mechanisms support informational and socially normative conformity
Source: PLoS Biol. 2022 Mar 3;20(3):e3001565. doi: 10.1371/journal.pbio.3001565 (PMC8893340; doi:10.1371/journal.pbio.3001565)
Supplement: S2 Text — dACC, dorsal anterior cingulate cortex. (DOCX) [file pbio.3001565.s002.docx]

**S2 Text**

**dACC actvity on observation trials**


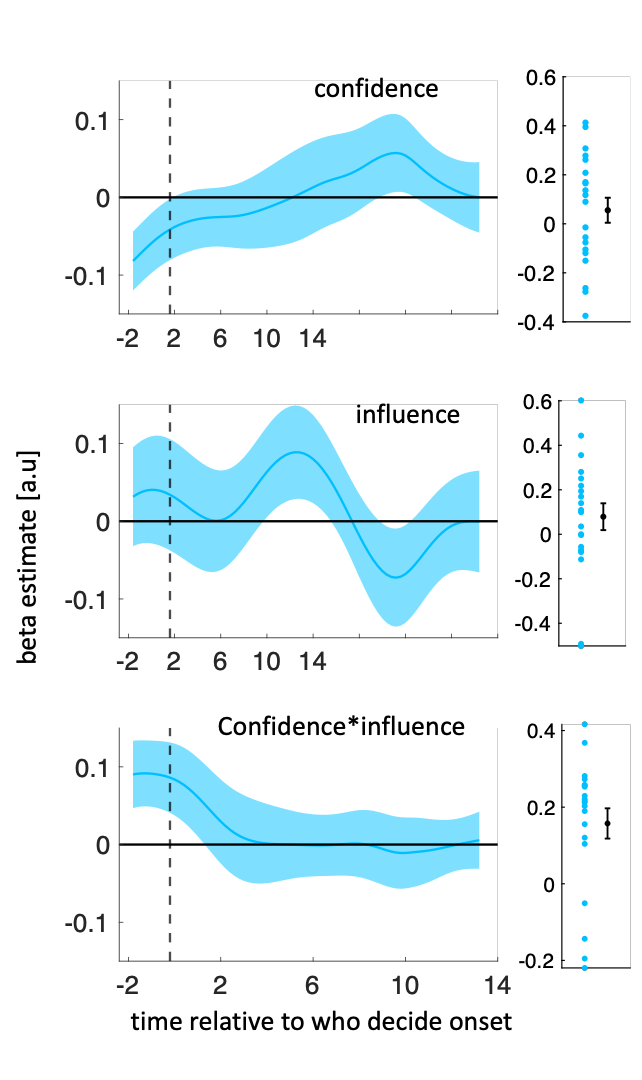


Figure S3: Only for the human condition, we computed response of the dACC to confidence (top), influence (middle), and their interaction (bottm) when it was announced that the partner would have to announce the revised estimate (See Figure 1). Unlike revise trials where the participants announced the revised decision, in these trials there was no effect of any of the variables on the dACC activity on the observe trials (panel A), the activity. The right panels show corresponding single subject estimate using leave-one-out procedure explained in the main text. Data and codes to recreate the figure are available at <https://github.com/alimahmoodia/Reciprocity_Data/tree/main>.
