# Supplementary material for: Distinct neurocomputational mechanisms support informational and socially normative conformity
Source: PLoS Biol. 2022 Mar 3;20(3):e3001565. doi: 10.1371/journal.pbio.3001565 (PMC8893340; doi:10.1371/journal.pbio.3001565)
Supplement: S3 Text — dACC, dorsal anterior cingulate cortex. (DOCX) [file pbio.3001565.s003.docx]

**S3 Text**

**Bayesian statistical analysis of the effect of informational and normative conformity in the dACC**

One challenge of interpreting the null result in the computer condition is that it might be due to lack of statistical power of the fMRI data set and not due to a lack of an effect. To this aim, we employed Bayesian statistics because a frequentist PHPA would provide very little information over and above the p-value already reported(1,2). On the other hand, Bayes Factors are an established tool to assess whether the lack of a significant result is rather due to no effect (i.e. evidence of absence) or a lack of evidence (i.e. absence of evidence) (3). We therefore employed Bayesian statistics to examine the reliability of one key claim in our paper, namely the interaction between confidence and influence in the human condition on dACC. We computed Bayes Factors (BF) for our main results, in particular for the computer condition and all our non-significant results. We used the categorisation to interpret our BFs (4,5). Under this scheme, 1/10<BF_10_<1/3 indicates moderate evidence for the null hypothesis (evidence of absence) and 10>BF_10_>3 indicates moderate evidence for the alternative hypothesis. More extreme values of BF_10_ (i.e. BF_10_<1/10 or BF_10_>10) provide even stronger evidence for the null or the alternative hypothesis. BF_10_ values between 1/3 and 3 indicate that there is insufficient evidence to draw any conclusion (absence of evidence) which could be due to small sample size. Critically, Bayes Factors are an established statistical method to clarify whether a nonsignificant result is likely due to low power (absence of evidence) or a genuine lack of effect (evidence of absence).

Using this procedure, and consistent with our results reported in the submitted manuscript, we found moderate evidence for the effect of the interaction between confidence and influence in the human condition on dACC BOLD (Bayes Factor (BF_10_) = 3.9) which is a key result of our manuscript. Notably, we found evidence for the null hypothesis (absence of an interaction effect in the computer condition) for the computer condition (BF_10_ = .23). In the manuscript, we have shown that the effect of confidence and its interaction with influence are only significant in revision but not in observation trials. We therefore computed Bayes factors for confidence and its interaction with influence in observation trials to see if the lack of significance is due to insufficient evidence or whether there is evidence in favour of the null hypothesis. Interestingly, in both cases we found evidence for the null hypothesis (confidence, BF_10_ = .23, and confidence influence interaction, BF_10_ = .07). This suggests that the lack of a statistically significant neural signature of normative conformity in the computer condition is likely due to the lack of an effect rather than a limited sample size.

We note that we obtained rich subject-level data for fMRI analysis. Each scan run (4 in total) lasted on average 15 minutes (range: [12 to 17 min]) – generating 120 trials of behavioural data and 176-251 brain volumes for fMRI data.

1. Lenth RV. Post hoc power: tables and commentary. Iowa City Dep Stat Actuar Sci Univ Iowa. 2007;1–13.

2. Yuan K-H, Maxwell S. On the post hoc power in testing mean differences. J Educ Behav Stat. 2005;30(2):141–67.

3. Keysers C, Gazzola V, Wagenmakers E-J. Using Bayes factor hypothesis testing in neuroscience to establish evidence of absence. Nat Neurosci. 2020;23(7):788–99.

4. Jeffreys H. The theory of probability. OUP Oxford; 1998.

5. Lee MD, Wagenmakers E-J. Bayesian cognitive modeling: A practical course. Cambridge university press; 2014.
