# Supplementary material for: Distinct neurocomputational mechanisms support informational and socially normative conformity
Source: PLoS Biol. 2022 Mar 3;20(3):e3001565. doi: 10.1371/journal.pbio.3001565 (PMC8893340; doi:10.1371/journal.pbio.3001565)
Supplement: S4 Text — dACC, dorsal anterior cingulate cortex; ROI, region of interest. (DOCX) [file pbio.3001565.s004.docx]

**S4 Text**

**Robustness of our dACC ROI analysis:**

The dACC is an area with enormous functional heterogeneity. Its functional heterogeneity has evoked a great deal of discussion in the scientific community (See Heilbronner and Hayden 2016, Silvetti et al. 2013, Kolling et al. 2016a, Shenhav et al. 2016, Kolling et al. 2016b). Therefore, we chose a subregion of this area which was previously reported to be involved in change of mind (Fleming et al. 2018). It is of great importance to show that our results are robust and not dependent on our specific ROI selection. We therefore defined three additional dACC ROIs according to other studies which reported the involvement of the dACC in social change of mind. We defined an ROI around the peak activity (MNI coordinate [-1 8 54], r = 10mm) of a previous study (Qi et al. 2018) which found the involvement of the dACC in social change of mind (henceforth Qi et al.). We also defined an ROI based on a previous meta-analysis (ref 8 in the main text) around MNI coordinate ([ 8 18 46] r = 10mm), (henceforth Wu et al.). Finally, we combined all three ROIs (our original ROI based on Fleming et al. 2018, the ROI obtained from Qi et al. 2018, and the ROI obtained from Wu et al. 2016). We have plotted the key time courses (confidence in red and its interaction with influence in blue). As indicated by the plots, the responses of the different ROIs to confidence and its interaction with influence are closely similar to each other (all significant, p<.05). It should be noted that our ROI does not overlap with the other two ROIs. We therefore believe that our results are robust across different dACC ROIs which were implicated in the process of social change of mind.

Figure S4: We created three more dACC ROIs and found that in all ROIs confidence and its interaction with influence had a significant effect on BOLD activity. Data and codes to recreate the figure are available at <https://github.com/alimahmoodia/Reciprocity_Data/tree/main>.
