# Supplementary material for: Distinct neurocomputational mechanisms support informational and socially normative conformity
Source: PLoS Biol. 2022 Mar 3;20(3):e3001565. doi: 10.1371/journal.pbio.3001565 (PMC8893340; doi:10.1371/journal.pbio.3001565)
Supplement: S5 Text — dmPFC, dorsomedial prefrontal cortex; ROI, region of interest; TPJ, temporoparietal junction. (DOCX) [file pbio.3001565.s005.docx]

**S5 Text**

**Robustness of our dmPFC and TPJ ROI analysis:**

Our TPJ and dmPFC masks were based on independent connectivity-based parcellations of the human brain (1)(2). As the social neuroscience literature is vast, this time – rather than identifying closely related studies – we created control masks using Neurosynth (3). For the dmPFC, we created a spherical mask (r = 10mm) around the peak MNI coordinate [2 46 38] returned by a forward search for “dmPFC”. For the TPJ, we combined spherical masks (r = 7mm) return by forward searches for “right TPJ” and “left TPJ” (MNI coordinates [58 -50 14], [-58 -54 18]). For both new masks, the TPJ and dmPFC responses to influence at the time of revision observation was similar to that seen for original ROIs (see figure below) and both set of responses were statistically significant (p<.05) (See Supplementary Material).


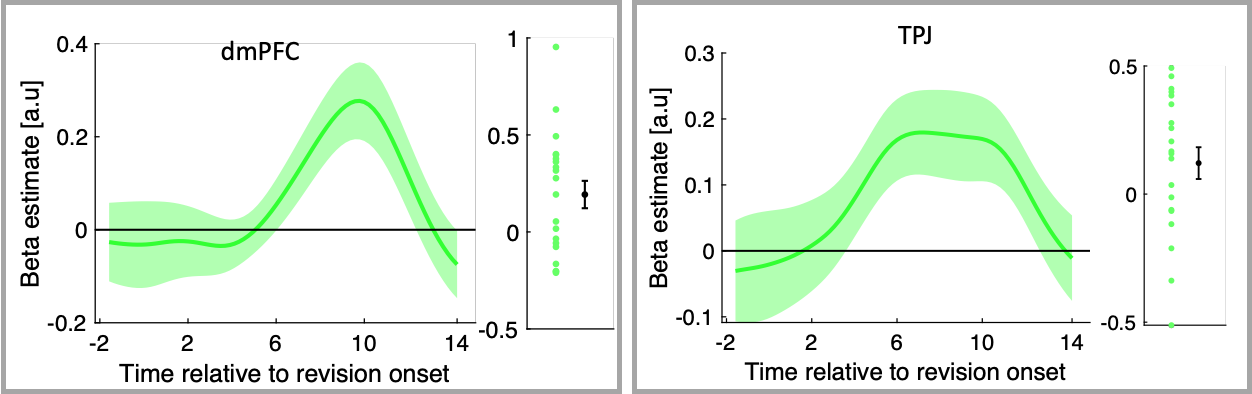


Figure S5: At the time of revision presentation, both dmPFC (left) and TPJ (right) encoded normative factor in the human condition when we repeated our analysis using independent ROIs created from neurosynth. Data and codes to recreate the figure are available at <https://github.com/alimahmoodia/Reciprocity_Data/tree/main>.

1. Sallet J, Mars RB, Noonan MP, Neubert F-X, Jbabdi S, O’Reilly JX, et al. The organization of dorsal frontal cortex in humans and macaques. J Neurosci. 2013;33(30):12255–74.

2. Mars RB, Sallet J, Schüffelgen U, Jbabdi S, Toni I, Rushworth MF. Connectivity-based subdivisions of the human right “temporoparietal junction area”: evidence for different areas participating in different cortical networks. Cereb Cortex. 2012;22(8):1894–903.

3. Yarkoni T, Poldrack RA, Nichols TE, Van Essen DC, Wager TD. Large-scale automated synthesis of human functional neuroimaging data. Nat Methods. 2011;8(8):665–70.
