# Supplementary material for: Distinct neurocomputational mechanisms support informational and socially normative conformity
Source: PLoS Biol. 2022 Mar 3;20(3):e3001565. doi: 10.1371/journal.pbio.3001565 (PMC8893340; doi:10.1371/journal.pbio.3001565)
Supplement: S6 Text — dACC, dorsal anterior cingulate cortex; dmPFC, dorsomedial prefrontal cortex. (DOCX) [file pbio.3001565.s006.docx]

**S6 Text**

**Connectivity analysis between dmPFC and dACC:**


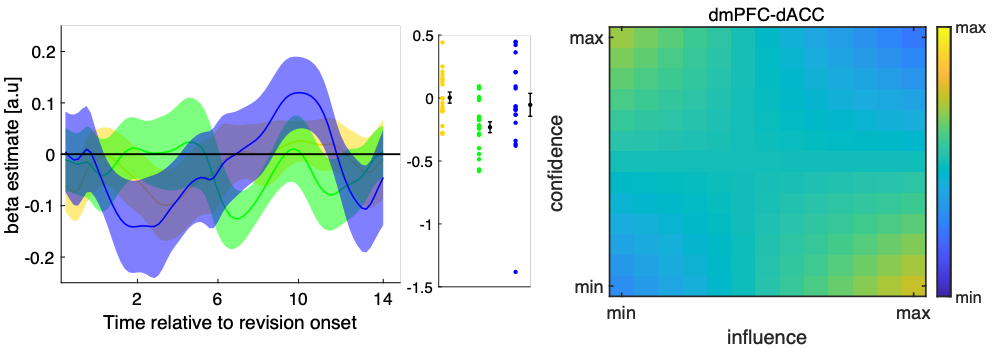


Figure S6: Psychophysiological interaction analysis of ROI activity time courses. Left, Traces are coefficients from a GLM in which we predicted dACC activity from the interaction between dmPFC activity and (1) confidence (yellow), (2) influence (green) and (3) the interaction between confidence and influence (blue) – while controlling for the main effect of each term. Middle, single subject estimate of the activity time course of the interaction between dmPFC and confidence (yellow), influence (green) and their interaction (blue) using leave-one-out procedure explain the methods. Right, Visualisation of dmPFC-dACC connectivity. Hotter colours indicate greater dmPFC-dACC connectivity as a function of variation (in z-score units) in influence (x-axis) and confidence (y-axis). dmPFC-dACC connectivity was estimated using group-level coefficients averaged across a time window from 2 s to 3 s. Data and codes to recreate the figure are available at <https://github.com/alimahmoodia/Reciprocity_Data/tree/main>.
