# Supplementary material for: Distinct neurocomputational mechanisms support informational and socially normative conformity
Source: PLoS Biol. 2022 Mar 3;20(3):e3001565. doi: 10.1371/journal.pbio.3001565 (PMC8893340; doi:10.1371/journal.pbio.3001565)
Supplement: S7 Text — PPI, psychophysiological interaction. (DOCX) [file pbio.3001565.s007.docx]

**S7 Text**

**Psychophysiological interaction comparison between human and computer conditions:**

We conducted a new Psychophysiological interaction analysis between TPJ and dACC at the time of revision in which we included all human and computer conditions and a new variable for condition (human or computer). We tested whether the interaction term that we reported in the main text (Figure 5) is quantitatively different between human and computer conditions. We found that there was a significant difference between the four-way interaction of condition and all variables of interests: confidence, influence and TPJ activity (Wilcoxon sign-ranked test, p=.01) (Figure S5).


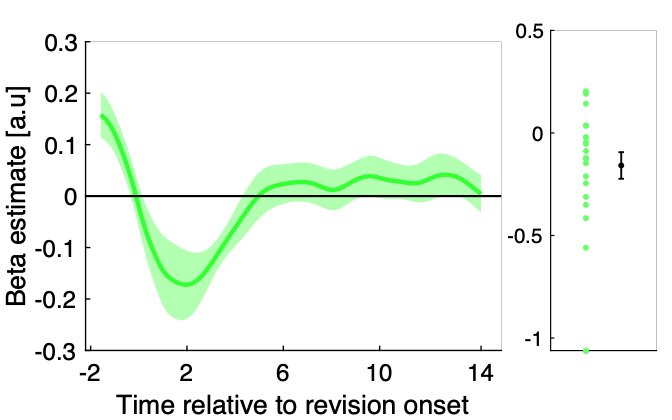


Figure S7: Psychophysiological interaction analysis of ROI activity time courses. Traces are coefficients from a GLM in which we predicted dACC activity from the interaction between TPJ activity and (1) confidence, (2) influence and (3) the interaction between confidence and influence, and condition– while controlling for the main effect of each term (confidence, influence, TPJ activity, and condition). The curve shows the four ways interaction between confidence, influence, TPJ activity and condition meaning that the connectivity is significantly lower in the human condition than in the computer condition. The right panel shows single subject estimate of the activity time course on the left using leave-one-out procedure explain the methods. Data and codes to recreate the figure are available at <https://github.com/alimahmoodia/Reciprocity_Data/tree/main>.
