# Supplementary material for: Distinct neurocomputational mechanisms support informational and socially normative conformity
Source: PLoS Biol. 2022 Mar 3;20(3):e3001565. doi: 10.1371/journal.pbio.3001565 (PMC8893340; doi:10.1371/journal.pbio.3001565)
Supplement: S8 Text — (DOCX) [file pbio.3001565.s008.docx]

**S8 Text**

**Exploratory whole-brain analysis:**

Finally, for completeness, we performed an exploratory whole-brain analysis in which we searched for neural correlates of our variables of interest (GLM1). We modelled the task events shown in Figure 1 as separate condition regressors and included separate condition regressors for human and computer blocks (note that some events only happen every other trial). We parametrically modulated a subset of the condition regressors as follows: the periods during which participants made their estimate (visual estimate) and indicated their confidence (confidence rating) were parametrically modulated by confidence; the period during which participants revised their estimate (revision) was parametrically modulated by confidence, the influence that the partner had exerted over participants on the previous trial, their interaction, the degree of revision, the angular distance between the participant’s own and the partner’s first estimate and the angular distance between participants’ revised and the partner’s first estimate; and the period during which participants observed the partner’s revised estimate (revision observation) was parametrically modulated by the influence that participants had exerted over the partner on the current trial.

Here we focus on the whole-brain equivalents of the ROI analyses reported above – see Table A for the full set of whole-brain results. At the time of the visual estimate, the whole-brain analysis identified a negative effect of confidence in the precuneus across human and computer conditions (Figure S8, peak coordinates [6 -48 50], k = 129, $t_{peak}$( (19) = 4.93, p < .0001). At the time of revision (revision trials only), and consistent with the ROI analysis, the whole-brain analysis identified a positive effect of confidence in a cluster encompassing dACC across human and computer conditions (Figure S9A; peak coordinates [2 -16 42], k = 59, $t_{peak}$( (19) = 6.26, p <.0001). Further, there was a positive effect of amount of revision in ventromedial prefrontal cortex (vmPFC) (Figure S9B peak coordinates [-10 44 -10], k = 26, $p_{FWE}<.05$). Finally, we note that the whole-brain analysis did not identify any clusters that tracked influence or its interaction with confidence in the human condition, nor any clusters that tracked the difference between the human and computer conditions at any of the events of interest.


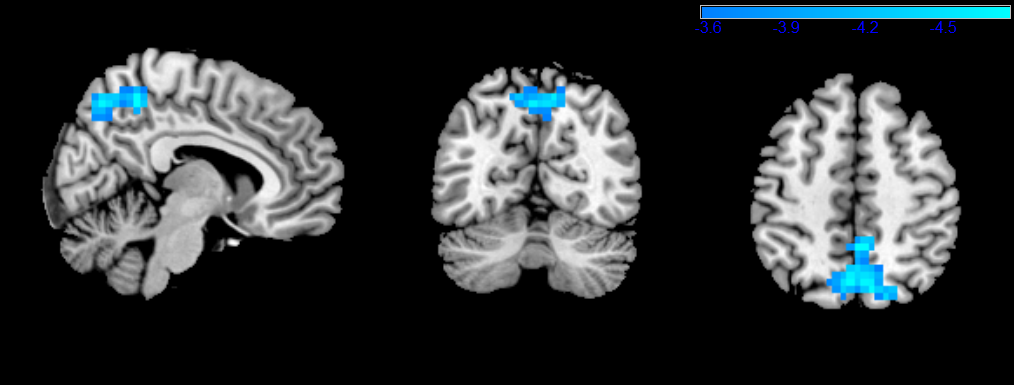


Figure S8: At whole brain, activity of precuneus at the time of first estimate (t2) was significantly negatively modulated by the confidence. Threshold at p<.05, FEW corrected for multiple comparisons, cluster definding threshold p<.0001. Data and codes to recreate the figure are available at <https://github.com/alimahmoodia/Reciprocity_Data/tree/main>

Figure S9: Exploratory whole-brain analysis. Aggregated across both conditions, dACC and vmPFC clusters were modulated by participants’ confidence (left) and their revision toward their partner’s estimate (right) at the time of Revision (t5). Clusters are significant at P < 0.05, FWE-corrected for multiple comparisons, with a cluster-defining threshold of P < 0.001, uncorrected. Data and codes to recreate the figure are available at <https://github.com/alimahmoodia/Reciprocity_Data/tree/main>.

| **Event** | **Parametric modulator** | **contrast** |
| --- | --- | --- |
| Estimate | Confidence | Human and computer versus all |
| Confidence rating | Confidence | Human and computer versus all |
| Revision | Confidence | Human and computer versus all |
| Revision | Influence | Human versus all and human versus computer |
| Revision | Influence* confidence | Human versus all and human versus computer |
| Revision | Amount of revision | Human versus all and human versus computer |
| Revision | Angular distance with partner before revision | Human and computer versus all |
| Revision | Angular distance with partner after revision | Human and computer versus all |
| Revision observation | Influence | Human versus all and human versus computer |

Table A: different whole brain contrasts which were tested at different event onsets.
