# Supplementary material for: Distinct neurocomputational mechanisms support informational and socially normative conformity
Source: PLoS Biol. 2022 Mar 3;20(3):e3001565. doi: 10.1371/journal.pbio.3001565 (PMC8893340; doi:10.1371/journal.pbio.3001565)
Supplement: Attachment — Submitted filename: Response to Reviewer.docx [file pbio.3001565.s009.docx]

Reviewer #1:

In this paper, the authors reported an fMRI study aiming to disentangling the neural substrates of "informational" vs. "normative" social conformity (or "social change of mind"). To this end, they developed a novel behavioral task by combining a visual perception task with social influence manipulation. The authors statistically dissociated informational and normative conformity by regressing the degree of behavioral changes on to participants' confidence of their own performance (i.e., informational conformity) and how much the influencer was influenced by the participants on separate trials (i.e., normative conformity). Neurally, the authors found that informational conformity was associated with dACC activity, regardless of whether the "information" was from a human or a computer agent; normative conformity also appeared to be correlated with dACC activity, but only when the confidence level was low and when the information was from a human agent. Normative information seems to be encoded in the social-called mentalizing network (e.g., dmPFC and TPJ) and conveyed to dACC via functional connectivity.

Overall, I enjoy reading this well-written paper. The behavioral task and statistical analysis offered a novel approach to quantify and dissociate informational and normative conformity (or social influence). The use of a computer agent condition was a clever way of demonstrating the social specificity of "normative" conformity. The fMRI analysis was guided by logically sound hypothesis and theorization. I have a few comments and suggestions.

We thank the reviewer for the careful review. We were happy to hear that that the reviewer found our behavioural and statistical analysis novel and our fMRI analysis approach logically sound. In the revised manuscript we have added further analyses and discussion points to address the reviewer’s comments. In the following, we separately address each comment/suggestion by the reviewer.

1.      The fMRI sample size (N = 20) is somewhat concerning. I urge the authors carry out post-hoc power analysis and report the achieved power of their various analysis (both behavioral and neural). This should be relatively straightforward because the fMRI results were primarily based on ROI analysis, which can be treated in the same way as behavioral data. This is particularly relevant because in some regression models the authors included multiple interaction terms. Was the number of observations sufficient for such a large number of predictors?

We thank the reviewer for raising this issue and for suggesting a post hoc power analysis (PHPA). We completely agree with the reviewer that additional analyses are useful to further assess robustness of our results. To this end, we employed Bayesian statistics because a frequentist PHPA would provide very little information over and above the p-value already reported(1,2). In particular, we computed Bayes factors for our main results which allows us to assess the strength of the evidence in favour of a significant result and assess whether the lack of a significant is a genuine null effect (i.e. evidence of absence) or a lack of evidence (i.e. absence of evidence)(3). We used the categorisation to interpret our BFs (4,5). Under this scheme, 1/10 < BF_10_ < 1/3 indicates moderate evidence for the null hypothesis (evidence of absence) and 10 > BF_10_ > 3 indicates moderate evidence for the alternative hypothesis. More extreme values of BF_10_ (i.e. BF_10_ < 1/10 or BF _10_ >10) provide even stronger evidence for the null or the alternative hypothesis. BF_10_ values between 1/3 and 3 indicate that there is insufficient evidence to draw any conclusion (absence of evidence), which could be due to small sample size.

These new analyses supported the main conclusions of our manuscript. In particular, we found moderate evidence for the effect of the interaction between confidence and influence in the human condition on dACC BOLD (Bayes Factor (BF_10_) = 3.9). Further, we found evidence for the null hypothesis (absence of an interaction effect) for the computer condition (BF_10_ = .23). This result shows that the lack of a statistically significant neural signature of normative conformity in the computer condition is likely due to the lack of an effect rather than limited sample size. Another key result of our manuscript was that the effects of confidence and its interaction with influence were significant in revision trials only and not in observation trials. Critically, the Bayesian analysis showed that the null results for observation trials were most likely due to the lack of an effect rather than limited sample size (confidence, BF_10_ = .23, and confidence influence interaction, BF_10_ = .07).

We have now mentioned these Bayesian analyses to the main text (lines 313-314) and have explained this procedure in detail in the Supplementary Material (lines 7-37).

Finally, we highlight that we obtained rich subject-level data for fMRI analysis. Each scan run (4 in total) lasted on average 15 minutes (range: [12 to 17 min]) – generating 120 trials of behavioural data and 176-251 brain volumes for fMRI data. We have now clarified this aspect of our study in the Supplementary Material (lines 38-40).

2.      The authors simulated multiple agents, who behaved differently. How quickly and clearly did the participants figure out such difference? Whether and how did the process of learning the agents' preferences influence the two forms of conformity?

We thank the reviewer for raising this interesting question. To address this question, we computed – for each participant and each condition (i.e. susceptible and insusceptible human and computer blocks) – the average revision in a sliding window of 5 trials which moved by 1 trial at each step (see new Figure 2D below). In the human blocks, statistical analysis showed that participants significantly changed their revision in response to the susceptible and insusceptible partners from the very first to the very last trials. Interestingly, while there was no difference between susceptible and insusceptible partners in early trials (Wilcoxon signed-rank test W = 598 p = .4), participants revision toward the susceptible partner was significantly higher than their revision toward the insusceptible partners in late trials (Wilcoxon signed-rank test W = 1302 p = .008).

We also analysed these temporal dynamics separately for the susceptible and insusceptible human partners: revision increased for the susceptible human partner (Wilcoxon sign-rank test W = 643, p = .04) and decreased for the unsusceptible human partner (Wilcoxon sign-rank test W = 1211, p = .03). None of these effects were found in the computer blocks. We have added this analysis to the main text (lines 216-237) and as Figure 2D.

3.      Related to Point 2, I wonder if it was possible that the susceptible agent was treated less seriously by the participants, regardless of the participants' confidence of their own performance. This effect, if existed, would be different from the Confidence * Influence interaction that the authors considered. The assumption underlying the latter interaction (Confidence * Influence) was that the participants saw both agents as equally informative and would follow them more if the participants were less confident of their own performance. However, the interaction effect I pointed out relies on a complementary hypothesis, namely the participants treat the susceptible agent as less valuable, less independent a source of information, therefore conform to their performance less, even when the participants are not confident of their own performance.

To address this point, for the human condition and for each participant, we computed the average revision toward each partner separately for high and low confidence. This analysis supported our previously reported results – with participants overall being more influenced by the susceptible partner – but also showed that influence varied with confidence for both partners (see figure below). In other words, participants did not disregard the susceptible partner and the behavioural difference between the two partners are likely to reflect normative concerns. We thank the reviewer for prompting this control analysis which we have now added to the main text (lines 208-214) and as Figure 2C.

4.      If I understand the model correctly, the model seems to only account for the agent's conformity tendency in the most recent trial. How stochastic was the agent's conformity tendency? Would it be useful to take into account more trials into the agent's history?

There were some degrees of stochasticity in the partner’s choice. As described in the methods, the partners’ second estimate was generated using the following algorithm. In the insusceptible blocks, participants’ influence over their partner was chosen randomly from a uniform distribution on the interval [0, 0.2]. For the susceptible partner, participants’ influence was chosen with a probability of 0.5 from a uniform distribution on the interval [0.7, 1], with a probability of 0.2 from a uniform distribution on the interval [0.3, 0.7], and with a probability of 0.3 from a uniform distribution on the interval [0, 0.3].

To address the reviewer’s question, we extended our model ‘lmm2’ in the main text to contain the influence that participants exerted on their partners in the last four trials. In other words, we predicted revision by confidence in the current trial, influence in the last four trials where the partner made the revision and also the interaction between confidence in the current trial and influence in the previous trial. Consistent with our previous model, confidence in the current trial (parameter estimate±95%CI = -.26±.1, t(1433) = -5.4, p<.0001) and influence in the previous trial (parameter estimate±95%CI = .19±.12, t(1433) = 3.25, p=001) and their interaction (parameter estimate±95%CI = -.3±.18, t(1433) = -3.3, p=.001) significantly affected revision. However, none of the influences that participants exerted over their partners in the earlier trials had any statistically significant effect on revision (all p-values > .2). In other words, revision behaviour had a short memory – only taking into account the most recent trial – although we acknowledge that the lack of statistical significance beyond the most recent trial could be due to insufficient data. Because of the absence of statistical significance beyond the most recent trial we conducted our analysis by only including the most recent trial. We have now mentioned this analysis where we introduce our linear mixed effect models (lines 200-202).

5.      In this task, "normative" conformity was conceptualized as an obligation to reciprocate an agent's conforming behaviors. Were the participants explicitly told that their partners would see how much they (i.e., the participants) revised their performance? This public versus private dimension matters a lot in interpreting the results of the current study (see my related comment in the next point). This critical detail was not clear to me from the authors' description.

We apologise for not clearly mentioning this detail in our task and thank the reviewer for pointing out this important detail. In our instructions, we said “After making your second estimate, the first and second estimates will be presented to you and the other player”. This design was implemented to induce an obligation to reciprocate as mentioned by the reviewer. We now clarify this aspect in the main text (lines 135-138).

6.      Related to Point 5, I am not sure it is appropriate to label the normative conformity in this study as "normative". Did the participants perceive the obligation to reciprocate an agent's conforming behaviors as a norm? To me, this obligation would be more accurately described as some form of interpersonal loyalty or indebtedness. There was no group norm to conform to, nor was there social pressure to enforce it (as in Asch's classic design). It seems to me necessary to at least discuss the distinction between the cognitive processes underlying conformity to a group versus imitating one single model/exemplar. Another relevant dimension is private versus public revision of behaviors, which would have critical impact on the dissociation between normative versus informational conformity. Correctly situating the mode of social context in the current study in these various forms of social influence/conformity will help the readers interpret the findings and their significance.
A broader comment is that, the authors could use more self-reported data to validate their operationalization and strengthen some of their behavioral and neural results (e.g., Did the participants willingly or reluctantly revise their performance according to the performance of the susceptible agent? Did the participants accurately discern the difference between the susceptible and the insusceptible agents? Did the participants view the susceptible and insusceptible agents as equally informative?).

We thank the reviewer for bringing up this interesting point. We recognise that participants might not have found themselves obliged to conform to any norm per se – or at least we do not have any evidence for that. In our manuscript, we follow the convention of defining normative conformity as any type of conformity whose goal is not to maximise accuracy but some social construct (e.g., social approval)(6). In our task, from a pure accuracy point of view, participants’ influence on a partner should not have affected the degree to which they revised their own estimate towards the partner.

To better understand the revision process, we compared candidate objective and subjective drivers. First, we compared objective task performance with subjective experience thereof. In the human blocks, participants’ error did not differ between the susceptible and insusceptible partners (susceptible mean: 56° insusceptible mean 57.5°, Wilcoxon sign-rank test W = 1071, p = .25). However, when we looked at subjective ratings (at the end of each block, participants estimated their own and their partner’s performance on a scale from 1 to), we found that participants rated their own performance as lower in the insusceptible than the susceptible blocks (see Figure below; Wilcoxon sign-rank test W = 678, p = .02). In addition, participants rated their own performance relative to that of their partner as lower in the insusceptible than the susceptible blocks (insusceptible: Wilcoxon sign-rank test W = 326, p = .02; susceptible: Wilcoxon sign-rank test W = 483, p = .68).

An informational account of conformity suggests that the participants should have revised more toward the insusceptible than the susceptible partner: they perceived their own performance as worse in the insusceptible block and worse than the insusceptible partner. In other words – in support of a normative account – participants did the exact opposite of what one would have expected from a pure accuracy point of view. We highlight that there was no pressure – other than social – to change the revision behaviour according to the partner type.

Finally, in support of our social manipulation being salient, we found during debriefing of the fMRI participants that all of them noticed that one human partner was more influenced by them than the other.

We have now included the rating analysis in the main text (line 238-253 and figure 2E) and discussed it in the discussion lines (405-410). We have also mentioned the debriefing procedure in the Supplementary Material (line 2-5).

7.      Figure 5D shows an interesting interactive effect of confidence and influence on TPJ-dACC connectivity: when confidence was low, the effect of influence had a strong positive effect on the connectivity; however, when confidence was high, influence seemed to have a negative effect on the connectivity (i.e., not only weaker positive effect, but actually reverse). Did the behavioral interaction (Figure 2) exhibited a similar pattern? It would be helpful to illustrate the behavioral effect using the heatmap (for easier comparison with the neural effect). If, indeed, the behavioral data shows a similar pattern of interaction, I wonder how the authors would interpret the reversal. Perhaps when the participants were extremely confidence of their own performance, they would devalue the performance of the agent who always follow them (i.e., more influenced by them).

We apologise for any misunderstanding about the connectivity plot. This heatmap is using z-scores which means that the min and max values do not reflect opposite signs. In fact, all values are negative (as indicated by the negative three-way interaction in Figure 5D). We now clarify this important point in the figure caption (line 353).

Following the reviewer’s suggestion, we have now performed a similar visualisation for the behavioural data. In line with the results of our linear model, we find that revision is (A) highest when confidence is low and influence is high and (B) lowest when confidence is high and influence is low (see Figure below). We have now added this visualisation to the main text (lines 202-208) and as Figure 2B.

8.      The task the authors developed did not rely on binary choice. Nevertheless, it would still be useful to discuss the recent advances in the understanding of the cognitive basis of social influence afforded by studies adopting speeded binary choice tasks and drift-diffusion modeling. For example, there was a paper showing how personal and social information are integrated into decision making about rewards (Tump et al., 2020, DOI: <https://doi.org/10.1126/sciadv.abb0266>), which seems to me particularly relevant to the current study. Other examples are: Germar et al., 2013, DOI: <https://doi.org/10.1177/0146167213508985>; Son et al., 2019, <https://doi.org/10.1038/s41598-019-48050-2>; Yu et al., 2021, <https://doi.org/10.1016/j.cognition.2021.104641>

We thank the reviewer for brining our attention to these highly relevant studies.

The Tump et al. study demonstrated that confidence is indeed a very reliable indicator of informational conformity. They found that, when people are confident in their private decision, they seek less social information and are keen to persuade others to follow them by responding quickly. By establishing the relationship between social information seeking and confidence, this finding strengthens the motivation for our design – namely that any revision towards a partner’s opinion that is directly proportional to one’s own confidence is an instance of informational conformity. We now cite the Tump et al study (lines 63-66 and line 159) when making this connection.

In addition, we now discuss this broader set of studies in the Discussion (lines 465-483):

Several studies have examined social influence broadly construed – including conformity, emulation, compliance and imitation – across multiple domains. These domains include perceptual decision-making (7,8) ; value-based decision-making (9,10) ; object preference (11,12); moral decision-making (13); charitable giving (14); and social punishment(15). . However, with the exception of one study (14), these studies did not include a non-social control similar to the computer condition in the current study. The exclusive use of human-human interactions means that these studies cannot easily isolate normative aspects of social influence. Our study goes beyond this earlier work in this respect and could pave the way for future studies of the behavioural and neural basis of different forms of social influence.

A number of the aforementioned studies used drift diffusion modelling (DDM) to assess how social and private information were combined into a decision (7,8,13,15). Two studies (8,15) presented the social information before the subjects had made their own private decision; one study(7) had the subjects make their private decision first and a final study (13) completely separated the private and social decision stages in an emulation paradigm that did not measure conformity. Remarkably, all studies found that social information change the rate of evidence accumulation (drift rate). Our paradigm did not employ binary choice and therefore does not lend itself to a DDM analysis. However, the motivation of our design and our results are consistent with those of Tump et al in that confidence is the key factor determining informational conformity. Future research would benefit from combining a DDM approach our Human vs Computer design.

Reviewer #2:

Mahmoodi et al. investigated the neural basis of social and non-social change of mind. In doing so, they used the same task which they developed previously and beautifully demonstrated both informational (i.e., effect of confidence = non-social change of mind) and normative (effect of a normative reciprocity effect = social change of mind) influence. They also found that the ACC was a key region for both types of influence and further showed that, the connectivity between the dACC and two social brain regions (namely dmPFC and PJ, both of which tracked the degree of normative influence) increased when interacting with a human partner. I think the question is very interesting, the task was creative and appropriate for testing their hypotheses, and they found results consistent with their hypotheses. However, I have several concerns which should be addressed before I recommend the paper for publication.

We thank the reviewer for the careful review. We were happy to hear that that the reviewer found the research question interesting. We have added further analyses and discussion points to address the reviewer’s comments. In the following, we separately address each comment/suggestion by the reviewer.

Major
1.    How was the fMRI sample size (n=20) determined? In general, n=20 is not considered to be many for any fMRI study with the current standard. Also, the present study used a 1.5 T scanner, which gives a lower S/N ratio than a 3.0 T scanner typically used in the field.

We determined our sample size based on previous fMRI studies related to ours – including Fleming et al. 2018 with 20 participants(16), Qi et al. 2018 with 23 participants(17), and Park et al. 2017 with 24 participants(18). We acknowledge that we are on the lower side of this range, but we are not far from the typical sample size in the field. In fact, the median number of participants in fMRI studies which were published in 2018 in high-ranking journals was 24(19). We therefore believe that our sample size is within an acceptable range for the field.

One common concern in the field is that non-significant results are due to small sample rather than the absence of an effect. To directly address this issue – and also in response to reviewer 1 (point 1)– we have now conducted Bayesian statistical analysis to show that the non-significant results reported in our manuscript were not due to small sample size. In particular, we computed Bayes factors for our main results which allows us to assess the strength of the evidence in favour of a significant result and assess whether the lack of a significant is a genuine null effect (i.e. evidence of absence) or a lack of evidence (i.e. absence of evidence)(3). We used the categorisation to interpret our BFs (4,5). Under this scheme, 1/10 < BF_10_ < 1/3 indicates moderate evidence for the null hypothesis (evidence of absence) and 10 > BF_10_ > 3 indicates moderate evidence for the alternative hypothesis. More extreme values of BF_10_ (i.e. BF_10_ < 1/10 or BF _10_ >10) provide even stronger evidence for the null or the alternative hypothesis. BF_10_ values between 1/3 and 3 indicate that there is insufficient evidence to draw any conclusion (absence of evidence), which could be due to small sample size.

These new analyses supported the main conclusions of our manuscript. In particular, we found moderate evidence for the effect of the interaction between confidence and influence in the human condition on dACC BOLD (Bayes Factor (BF_10_) = 3.9). Further, we found evidence for the null hypothesis (absence of an interaction effect) for the computer condition (BF_10_ = .23). This result shows that the lack of a statistically significant neural signature of normative conformity in the computer condition is likely due to the lack of an effect rather than limited sample size. Another key result of our manuscript was that the effects of confidence and its interaction with influence were significant in revision trials only and not in observation trials. Critically, the Bayesian analysis showed that the null results for observation trials were most likely due to the lack of an effect rather than limited sample size (confidence, BF_10_ = .23, and confidence influence interaction, BF_10_ = .07).

We have now mentioned these Bayesian analyses to the main text (lines 313-314) and have explained this procedure in detail in the Supplementary Material (lines 7-37).

Finally, we highlight that we obtained rich subject-level data for fMRI analysis. Each scan run (4 in total) lasted on average 15 minutes (range: [12 to 17 min]) – generating 120 trials of behavioural data and 176-251 brain volumes for fMRI data. We have now clarified this aspect of our study in the Supplementary Material (lines 38-40).

Regarding the use of a 1.5 T scanner, we agree with the reviewer that it has a lower signal-to-noise ratio than a 3.0 T scanner. However, we believe that this approach does not affect our manuscript as we are not seeking to segregate closely adjacent brain areas or precisely locate brain functions (e.g., as shown below, our dACC results generalise across slightly different anatomical masks).

2.    The main findings of the present study are based on the ROI analysis. The authors used a very specific ROI (their dACC ROI was exactly the same cluster reported in the previous study by Fleming et al. [2018]). The authors stated it is because the dACC "has consistently been linked to changes of mind in social (8) and non-social situations (1)." However, the dACC regions reported in refs 1 & 8 are different within the dACC (the dACC region related to social conformity is more anterior than the dACC region related to confidence). Furthermore, this strict ROI approach could lead us to misleading results. For example, although the current study showed that the same dACC area is sensitive to both social and non-social change of mind, it might well be that different regions within the dACC are associated with each of social and non-social changes of mind (and maybe overlaps in between).

We thank the reviewer for bringing up this important issue. We agree that it is possible that different neuronal populations (voxels) within the dACC might be involved in social and non-social aspects of change of mind. We feel that a complete voxel-wise mapping is beyond the scope of our study. Instead, we now acknowledge this point in the Discussion (lines 459-464) and – see our reply to point 3 –show that our results hold across different dACC masks.

3.    Related to the point above, the strict ROI approach could also increase the chance of finding false-positive results if there is a researcher's degree of freedom in terms of the way of selecting a ROI. I am not saying that the authors did cherry-picking, but since this study was not pre-registered, and there are many other seemingly valid ways of defining a specific dACC ROI (e.g., use a different study that reported dACC activity related to confidence, use a study that reported social conformity related dACC activity, use Neurosynth, etc.), it is important to demonstrate how robust the present finding is especially given the small fMRI sample size. For example, can you find dACC activation (related to confidence or interaction between confidence and influence) within more broadly defined dACC ROI (e.g., anatomically defined)?

We thank the reviewer for prompting us to test whether the results hold for different dACC specifications. Our ROI was based on a sub-region of dACC which has previously been shown to be involved in changes of mind, but we agree with the reviewer – especially given the vast literature on different accounts of the dACC (20–22) – to assess the degree to which our results depend on a particular dACC mask. To do so, we defined three additional dACC ROIs based on previous studies on the role of dACC in social changes of mind. First, we defined an ROI around the dACC peak activity (MNI coordinate [-1 8 54], r = 10mm) identified by Qi et al. (2018)(17). Second, we defined an ROI based around the dACC locus identified by the meta-analysis by Wu et al. (2018) ([ 8 18 46] r = 10mm)(23). Finally, we combined these two ROIs with our original ROI (i.e., the union of the three masks). Next, we repeated our key analysis of the impact of confidence (red) and its interaction with influence (blue) on dACC activity time courses across all four ROI masks (see below). Critically, the response profiles were highly similar across all four ROI masks. We therefore believe that our results are robust to the precise anatomical specification of dACC.

We have now included this analysis in the Supplementary Materials (lines 67-84 and Figure S2) and discuss it in the main text (lines 319-323).

4.    The same point applies to the TPJ and dmPFC ROIs.

Our TPJ and dmPFC masks were based on independent connectivity-based parcellations of the human brain(24,25). As the social neuroscience literature is vast, this time – rather than identifying closely related studies – we created control masks using Neurosynth. For the

dmPFC, we created a spherical mask (r = 10mm) around the peak MNI coordinate [2 46 38] returned by a forward search for “dmPFC”. For the TPJ, we combined spherical masks (r = 7mm) return by forward searches for “right TPJ” and “left TPJ” (MNI coordinates [58 -50 14], [-58 -54 18]). For both new masks, the TPJ and dmPFC responses to influence at the time of revision observation was similar to that seen for original ROIs (see figure below) and both set of responses were statistically significant (p<.05).

We again thank the reviewer for prompting us to demonstrate the robustness of our neural results. We have now added this analysis to the Supplementary Materials (lines 100-109 and Figure S3) and discuss it in the main text (lines 338-343).

5.    Regarding the dACC-TPJ/dmPFC connectivity, it is also important to demonstrate the difference in the connectivity between the human vs. computer conditions.

We have now conducted the dACC-TPJ/dmPFC connectivity analysis for the computer condition as well. Specifically, we predicted dACC activity from the interaction between TPJ/dmPFC activity and (1) confidence (yellow), (2) influence (green) and (3) the interaction between confidence and influence (blue) – while controlling for the main effect of each term (confidence, influence and TPJ/dmPFC activity). Critically, the connectivity pattern in the computer condition does not match the connectivity pattern observed in the human condition. In fact, none of the predictors of interest were significantly correlated with dACC activity. We have now included this analysis in the Supplementary Material (Figure S4) and discuss it in the main text Lines 373-375.

Minor:

6. As long as I understand, a total of 60 participants took part in the study, and 1/3of them performed the task inside the scanner. So, the fMRI data is based on 20 participants. I wonder if the data from all 60 participants were included in the behavioral analysis?

All behavioural data analyses include all 60 participants. We thank the reviewer for pointing out this lack of clarity which we have now rectified (lines 150-151).

7. What instruction did the participants receive about the computer condition? Did they know the computer's decision was more or less accurate (thus informative)?

Participants were simply told that they would interact either with a human being or a computer algorithm. Participants were not informed about the partners’ accuracy or strategy in either the human or the computer blocks. For transparency, we have uploaded our task instruction to open platform which can be found here: https://figshare.com/articles/dataset/Task_instruction/17280473

We have added this detail in the Methods lines 573-575.

We note – as the reviewer may also have had this issue in their mind – that participants did not find the computer partners to be better or worse than the human partners as revealed by rating data elicited at the end of each block (Wilcoxon signed-rank test W = 533, p = .93, ${BF}_{10}= .15$). This equivalence was objectively true as both human and computer partners were in reality generated using the same algorithm.

8. L383: It says "The first stimulus was presented for 30 ms." But, the figure 1a says it was presented for 25ms. Please clarify.

We thank the reviewer for pointing out to this error. We have now clarified in Figure 1a that the stimulus was presented for 30ms.

Reviewer #3:

The manuscript "Distinct neurocomputational mechanisms support informational and normative Conformity" continues a line of very interesting findings probing neurocomputational mechanisms of informational and normative conformity. In brief, the results suggest that the dACC tracks informational conformity towards both human and computer but it tracks normative conformity only when interacting with human. In addition, the dmPFC and TP track normative conformity, they are also functionally coupled with the dACC when interacting with humans. The findings are interesting, but authors have to clarify some details of the study:

We thank the reviewer for the careful review. We were happy to hear that that the reviewer found our findings interesting. We have added further analyses and discussion points to address the reviewer’s comments. In the following, we separately address each comment/suggestion by the reviewer.

1)      Line 117 states that twenty  participants participated in the fMRI  study (N = 20) and forty  participants participated in a  behavioral study  (N = 40).

(a)     What was the reason to have a relatively small sample size in the fMRI study?

We determined our sample size based on previous fMRI studies related to ours – including Fleming et al. 2018 with 20 participants(16), Qi et al. 2018 with 23 participants(17), and Park et al. 2017 with 24 participants(18). We acknowledge that we are on the lower side of this range, but we are not far from the typical sample size in the field. In fact, the median number of participants in fMRI studies which were published in 2018 in high-ranking journals was 24(19). We therefore believe that our sample size is within an acceptable range for the field.

In addition, as also mentioned in our responses to reviewer 1 (point 1) and reviewer 2 (point 1), we have now complemented our classical statistical analysis with Bayesian approaches and show that our key null effects (e.g., lack of neural encoding of normative factors in the computer condition) are due to the absence of an effect rather than sample size (absence of evidence). We therefore believe that sample size is not an issue for our results. We repeat our reply again here for ease of reference:

One common concern in the field is that non-significant results are due to small sample rather than the absence of an effect. To directly address this issue – and also in response to reviewer 1 (point 1)– we have now conducted Bayesian statistical analysis to show that the non-significant results reported in our manuscript were not due to small sample size. In particular, we computed Bayes factors for our main results which allows us to assess the strength of the evidence in favour of a significant result and assess whether the lack of a significant is a genuine null effect (i.e. evidence of absence) or a lack of evidence (i.e. absence of evidence)(3). We used the categorisation to interpret our BFs (4,5). Under this scheme, 1/10 < BF_10_ < 1/3 indicates moderate evidence for the null hypothesis (evidence of absence) and 10 > BF_10_ > 3 indicates moderate evidence for the alternative hypothesis. More extreme values of BF_10_ (i.e. BF_10_ < 1/10 or BF _10_ >10) provide even stronger evidence for the null or the alternative hypothesis. BF_10_ values between 1/3 and 3 indicate that there is insufficient evidence to draw any conclusion (absence of evidence), which could be due to small sample size.

These new analyses supported the main conclusions of our manuscript. In particular, we found moderate evidence for the effect of the interaction between confidence and influence in the human condition on dACC BOLD (Bayes Factor (BF_10_) = 3.9). Further, we found evidence for the null hypothesis (absence of an interaction effect) for the computer condition (BF_10_ = .23). This result shows that the lack of a statistically significant neural signature of normative conformity in the computer condition is likely due to the lack of an effect rather than limited sample size. Another key result of our manuscript was that the effects of confidence and its interaction with influence were significant in revision trials only and not in observation trials. Critically, the Bayesian analysis showed that the null results for observation trials were most likely due to the lack of an effect rather than limited sample size (confidence, BF_10_ = .23, and confidence influence interaction, BF_10_ = .07).

We have now mentioned these Bayesian analyses to the main text (lines 313-314) and have explained this procedure in detail in the Supplementary Material (lines 7-37).

Finally, we highlight that we obtained rich subject-level data for fMRI analysis. Each scan run (4 in total) lasted on average 15 minutes (range: [12 to 17 min]) – generating 120 trials of behavioural data and 176-251 brain volumes for fMRI data. We have now clarified this aspect of our study in the Supplementary Material (lines 38-40).

(b)     From the manuscript it not clear whether results of the  behavioral study  are included into the data analysis, one way or another.

All behavioural data analyses include all 60 participants. We thank the reviewer for pointing out this lack of clarity which we have now rectified (lines 150-151).

(c)     Did authors find a difference between "in-scanner" and "out of the scanner" behavior?

We repeated our LMM2 analysis (where we predicted revision by confidence and influence and their interaction) for “fMRI subjects only” and “behavioural subjects only”. The table below provides estimated effect size and test statistics. Critically, the estimated 95% confidence intervals overlap for the “in-scanner” and “out-out-scanner” settings.

| Regressors | Confidence | Influence | interaction |
| --- | --- | --- | --- |
| Human:  FMRI subjects | Beta±95%CI: -.19±.18  p-value = .04 | Beta±95%CI: .21±.2  p-value = .04 | Beta±95%CI: -.32±.31  p-value = .04 |
| Human: Behavioural subjects | Beta±95%CI: -.35±.12  p-value = 2e-9 | Beta±95%CI: .15±.13  p-value = .02 | Beta±95%CI: -.16±.22  p-value = .14 |
| Computer:  FMRI subjects | Beta±95%CI: -.36±.19  p-value = .0002 | Beta±95%CI: .04±.23  p-value = .73 | Beta±95%CI: -.07±.39  p-value = .61 |
| Computer:  Behavioural subjects | Beta±95%CI: -.38±.17  p-value = 6e-7 | Beta±95%CI: -.01±.13  p-value = .93 | Beta±95%CI: .02±.22  p-value = .8 |

We also extended our LMM2 to include a new variable to code for setting (fMRI, behaviour). The interaction of the setting with none of the variables of interest was significant. We therefore conducted our analysis with a simpler model which did not include the setting term. We have discussed this detail in Methods lines 606-609.

2)      How did the cover story exactly explain the computer choices? I.e. how did it explain computers' revisions (e.g. the algorithm and the goal of such revisions)?

Participants were simply told that they would interact either with a human being or a computer algorithm. Participants were not informed about the partners’ accuracy or strategy in either the human or the computer blocks. For transparency, we have uploaded our task instruction to open platform which can be found here: https://figshare.com/articles/dataset/Task_instruction/17280473

We have added this detail in the Methods lines 575-577.

3)      Line 425. From "Constructing computer partners" paragraph I got an impression that computer partners' choices differed from human partners' choices. At least, authors stated a different algorithm for the computer partners.  Can you give a reason for such differences?

We apologise for this lack of clarity and have now modified the text to clarify that the human and computer partners were constructed using exactly the same algorithm (lines 567-572).

4)      Can it be that in the observational trials participants simply lowered attention toward the screen? Such lack of attention perhaps could explain differential fMRI results for the revision and observation trials.

We agree that the participants might have lowered attention on observation trials and in that this in turn may have impacted the neural results (e.g., encoding of confidence and influence). However, we note that our main conclusions were based on revision and not observation trials and we believe that the fact that participants’ influence on the partners as revealed by observation trials had an impact on participants’ behaviour on revision trials shows that participants paid closely attention to the task events. We now briefly discuss this issue when presenting the results for the observation trials (lines 315-318).

5)      When making inferences about more than one parameter, you may use multiple comparison procedures to make inferences about the parameters of interest. Could you please comment the multiple comparison issue in the current study?

We thank the reviewer for bringing up this important issue. We have done Holm-Bonferroni correction whenever there was a multiple comparison problem. We also used adjusted degrees of freedom using Satterthwaite approximation to compute effective degrees of freedom which provides more accurate statistics. We have explained this issue in line 610-612 in the Methods.

6)      Authors treat the  "normative reciprocity effect" as the signature of  normative conformity. But according to other studies (e.g. Samuel J. Gershman, Hillard Thomas Pouncy, Hyowon Gweon, 2017) this behavioral phenomena can be understood as a form of informational social influence. To me it is still not clear if the  learning of the structure of social influence represents normative conformity.

We thank the reviewer for bringing up this interesting paper. Gershman and colleagues (2017) developed a model which accounted for humans’ social influence by inferring the hidden social structure among individuals based on their choice similarities. Their model is based on the following assumptions: “Each individual is assumed to belong to a latent group that parameterizes the distribution over choices. Due to the stochastic nature of choice, different individuals within the same group may not make exactly the same choices. The assignment of individuals to groups is unknown and must be inferred from observed data.”. In a nutshell, subjects infer to which group they belong based on the similarity between their own choices and those of others. Subjects are then more strongly influenced by those individuals who belong to the same group. As such, the model makes predictions about social influence in different group settings that go beyond social influence in purely dyadic situations. Because the model is based on Bayesian inference, it can be considered "rational" to some extent, for example, in the way it assigns individuals to groups. However, the model does not provide a “rational” explanation for why individuals assigned to the same group make similar choices. Instead, this is one of the assumptions on which the model is based. Therefore, the model does not provide an explanation of normative social influence by informational social influence.

Moreover, the model proposed by Gershman and colleagues does not take into account participants’ accuracy as there is no correct response in their paradigm (preference for movies). Accuracy is, however, crucial for the effect we report. Our central claim is that an agent which would be aiming to maximise accuracy would not revise less toward the insusceptible partner and more toward an susceptible partner when both partners have the same actual and perceived task performance. In fact, Figure 1E indicates that participants believe that their own performance is lower when they play with an insusceptible partner. Under informational social influence, participants should have revised more toward the insusceptible partner – the opposite of what we find. We believe that the model of Gershman and colleagues does not account for this experimental observation. We have now revised the main text to clarify that normative social influence in our task is very unlikely to reflect informational social influence (lines 405-410).

7)      Line 363 suggests that "humans can and do interact with non-human social partners via informational but not normative conformity".  The study address only one form of conformity - normative reciprocity effect. Will the results be replicated for other types of norms?

We thank the reviewer for prompting us to discuss this important issue. We have now added the following text to the Discussion (lines 489-495).

“It is important to note that reciprocity is only one among many well-known social norms. When we interact with others, we also wish to be included and accepted, be treated fairly, and have our right to privacy, dignity and agency respected(26). In choosing to focus on reciprocity, we were motivated by its longstanding history and the wide-spread consensus on this norm among different cultures(27). Future research should address the extent to which the same neurocomputational mechanisms support different social norms.”

8)      The text completely missing limitations of the study! Authors have to address this issue.

We thank the reviewer for prompting us to reflect on the limitations of our study and we have now added the following text to the Discussion (lines 496-509):

One limitation of our study is that we only studied the impact of two types of social influence on social changes of mind. However, various motives contribute to people’s social behaviour (28). Seeking elevated accuracy(29) is perhaps the most popularly recognized motive but only one among many other motives. Other motives include responsibility and regret (30), and equality(31). Future research could help determine whether and to what extent our participants were driven by these various motives.

Another limitation of our study is that our visual stimulation paradigm was not optimised for activating visual cortex. This aspect may explain why the whole-brain search did not identify social effects on sensory processing. A long-standing question in the literature of social conformity (32) is whether social information changes the brain processes at the level of sensation or at the level of cognition and decision-making. While numerous studies have sought to address this issue, the debate remains unresolved (11,33,34). One avenue for future research is combine our social paradigm which are known to drive visual activations in a reliable and retinotopically organised manner (e.g., high-contrast, rapidly-flickering Gabor patches).

References:

1.	Lenth RV. Post hoc power: tables and commentary. Iowa City Dep Stat Actuar Sci Univ Iowa. 2007;1–13.

2.	Yuan K-H, Maxwell S. On the post hoc power in testing mean differences. J Educ Behav Stat. 2005;30(2):141–67.

3.	Keysers C, Gazzola V, Wagenmakers E-J. Using Bayes factor hypothesis testing in neuroscience to establish evidence of absence. Nat Neurosci. 2020;23(7):788–99.

4.	Jeffreys H. The theory of probability. OUP Oxford; 1998.

5.	Lee MD, Wagenmakers E-J. Bayesian cognitive modeling: A practical course. Cambridge university press; 2014.

6.	Cialdini RB, Goldstein NJ. Social influence: Compliance and conformity. Annu Rev Psychol. 2004;55:591–621.

7.	Tump AN, Pleskac TJ, Kurvers RH. Wise or mad crowds? The cognitive mechanisms underlying information cascades. Sci Adv. 2020;6(29):eabb0266.

8.	Germar M, Schlemmer A, Krug K, Voss A, Mojzisch A. Social influence and perceptual decision making: A diffusion model analysis. Pers Soc Psychol Bull. 2014;40(2):217–31.

9.	Najar A, Bonnet E, Bahrami B, Palminteri S. The actions of others act as a pseudo-reward to drive imitation in the context of social reinforcement learning. PLoS Biol. 2020;18(12):e3001028.

10.	Charpentier CJ, Iigaya K, O’Doherty JP. A neuro-computational account of arbitration between choice imitation and goal emulation during human observational learning. Neuron. 2020;106(4):687–99.

11.	Izuma K, Adolphs R. Social manipulation of preference in the human brain. Neuron. 2013;78(3):563–73.

12.	Campbell-Meiklejohn DK, Bach DR, Roepstorff A, Dolan RJ, Frith CD. How the opinion of others affects our valuation of objects. Curr Biol. 2010;20(13):1165–70.

13.	Yu H, Siegel JZ, Clithero JA, Crockett MJ. How peer influence shapes value computation in moral decision-making. Cognition. 2021;211:104641.

14.	Chierchia G, Piera Pi-Sunyer B, Blakemore S-J. Prosocial influence and opportunistic conformity in adolescents and young adults. Psychol Sci. 2020;31(12):1585–601.

15.	Son J-Y, Bhandari A, FeldmanHall O. Crowdsourcing punishment: Individuals reference group preferences to inform their own punitive decisions. Sci Rep. 2019;9(1):1–15.

16.	Fleming SM, Van Der Putten EJ, Daw ND. Neural mediators of changes of mind about perceptual decisions. Nat Neurosci. 2018;21(4):617–24.

17.	Qi S, Footer O, Camerer CF, Mobbs D. A collaborator’s reputation can bias decisions and anxiety under uncertainty. J Neurosci. 2018;38(9):2262–9.

18.	Park SA, Goïame S, O’Connor DA, Dreher J-C. Integration of individual and social information for decision-making in groups of different sizes. PLoS Biol. 2017;15(6):e2001958.

19.	Szucs D, Ioannidis JP. Sample size evolution in neuroimaging research: An evaluation of highly-cited studies (1990–2012) and of latest practices (2017–2018) in high-impact journals. NeuroImage. 2020;221:117164.

20.	Heilbronner SR, Hayden BY. Dorsal anterior cingulate cortex: a bottom-up view. Annu Rev Neurosci. 2016;39:149–70.

21.	Shenhav A, Cohen JD, Botvinick MM. Dorsal anterior cingulate cortex and the value of control. Nat Neurosci. 2016;19(10):1286–91.

22.	Kolling N, Wittmann MK, Behrens TE, Boorman ED, Mars RB, Rushworth MF. Value, search, persistence and model updating in anterior cingulate cortex. Nat Neurosci. 2016;19(10):1280–5.

23.	Wu H, Luo Y, Feng C. Neural signatures of social conformity: A coordinate-based activation likelihood estimation meta-analysis of functional brain imaging studies. Neurosci Biobehav Rev. 2016;71:101–11.

24.	Sallet J, Mars RB, Noonan MP, Neubert F-X, Jbabdi S, O’Reilly JX, et al. The organization of dorsal frontal cortex in humans and macaques. J Neurosci. 2013;33(30):12255–74.

25.	Mars RB, Sallet J, Schüffelgen U, Jbabdi S, Toni I, Rushworth MF. Connectivity-based subdivisions of the human right “temporoparietal junction area”: evidence for different areas participating in different cortical networks. Cereb Cortex. 2012;22(8):1894–903.

26.	Eisenberger NI, Lieberman MD, Williams KD. Does rejection hurt? An fMRI study of social exclusion. Science. 2003;302(5643):290–2.

27.	Becker LC. Reciprocity. Routledge; 2014.

28.	El Zein M, Bahrami B, Hertwig R. Shared responsibility in collective decisions. Nat Hum Behav. 2019;3(6):554–9.

29.	Bahrami B, Olsen K, Latham PE, Roepstorff A, Rees G, Frith CD. Optimally interacting minds. Science. 2010;329(5995):1081–5.

30.	El Zein M, Bahrami B. Joining a group diverts regret and responsibility away from the individual. Proc R Soc B. 2020;287(1922):20192251.

31.	Mahmoodi A, Bang D, Olsen K, Zhao YA, Shi Z, Broberg K, et al. Equality bias impairs collective decision-making across cultures. Proc Natl Acad Sci. 2015;112(12):3835–40.

32.	Sherif M. A study of some social factors in perception. Arch Psychol Columbia Univ. 1935;

33.	Berns GS, Chappelow J, Zink CF, Pagnoni G, Martin-Skurski ME, Richards J. Neurobiological correlates of social conformity and independence during mental rotation. Biol Psychiatry. 2005;58(3):245–53.

34.	Falk EB, Berkman ET, Mann T, Harrison B, Lieberman MD. Predicting persuasion-induced behavior change from the brain. J Neurosci. 2010;30(25):8421–4.
