# Supplementary material for: Distinct neurocomputational mechanisms support informational and socially normative conformity
Source: PLoS Biol. 2022 Mar 3;20(3):e3001565. doi: 10.1371/journal.pbio.3001565 (PMC8893340; doi:10.1371/journal.pbio.3001565)
Supplement: Attachment — Submitted filename: Response to Reviewers.docx [file pbio.3001565.s010.docx]

Thank you for submitting your revised Research Article entitled "Distinct neurocomputational mechanisms support informational and socially normative conformity" for publication in PLOS Biology. I have now discussed your new version with other staff editors and with the Academic Editor. I am pleased to let you know that we are positive about your manuscript. However, before we can make a final decision about publication, we would like you to address the following points raised by the Academic Editor:

We thank the editor and the academic editor for positive assessment of our work. In the following, we separately address each comment/suggestion by the academic editor.

1) Reviewer 2 (point 5) asked to see the human vs. computer interaction of the PPI effect. Instead, you report that the effects in the computer condition are non-significant, whereas the human effect is significant. This is not the same as testing the interaction (<https://www.nature.com/articles/nn.2886>).

We apologise for missing the reviewer’s point. It now becomes clear that we misunderstood the reviewer’s point. In the revised manuscript, we have modified our connectivity analysis to contain a regressor for condition. Our analysis shows that the four-way interaction between condition and the other explanatory variables (confidence, influence, and TPJ activity) is in fact statistically significant (p = .01). We have replaced the old analysis with the new analysis in the supplementary material (Lines 154-170 Figure S5). We have also referred to this analysis in the main text. We thank the academic editor for drawing our attention to this.

2) The small fMRI sample size should be mentioned as limitation.

We have added the following text to the discussion where we discuss the limitations of the study lines 514-519:

Finally, we recognise that sample size (N = 20) for the number of participants in our fMRI experiment puts some limitation on conclusions that can be drawn from our findings. To address this issue, we used methods from Bayesian statistics to show that our negative findings (especially, the absence of a reciprocity effect in the computer condition) was not likely to be due to small sample size. We have made our data available to researchers interested in this paradigm and assessing normative conformity in future studies to help them plan their study sample size.

3) Line 618: “44 volumes of 2 mm slices”. “Volumes” is usually used to describe 3D images collected during one TR. Do you mean “slices” instead of “volumes” (i.e., “44 slices, 2 mm thick”)?

We apologise for this error and we thank the academic editor for pointing this out to us. We have now corrected the text as follow: “each volume contained 44 slices of 2 mm thickness”.

4) To avoid confusion, the text provided in the supplementary material (“Each scan run (4 in total) lasted on average 15 minutes (range: [12 to 17 min]) – generating 120 trials of behavioural data and 176-251 brain volumes for fMRI data. ”) should be part of the methods subsection “MRI data acquisition”.

We have added this information to the methods, section MRI data acquisition, Lines 627-628.

Please also make sure to address the following data and other policy-related requests.

5) Title: We would like to suggest a title that might be more appealing to a broad readership. We recommend:

"Distinct neurocomputational mechanisms support informational and social conformity."

or

"Distinct neurocomputational mechanisms support informational and socially normative conformity."

However, we would be happy to work with you on an alternative if you think our suggestions misrepresent your findings.

We have now replaced our title with “Distinct neurocomputational mechanisms support informational and socially normative conformity”.

6) Blurb: Please provide a blurb which (if accepted) will be included in our weekly and monthly Electronic Table of Contents, sent out to readers of PLOS Biology, and may be used to promote your article in social media. The blurb should be about 30-40 words long and is subject to editorial changes. It should, without exaggeration, entice people to read your manuscript. It should not be redundant with the title and should not contain acronyms or abbreviations. For examples, view our author guidelines: <https://journals.plos.org/plosbiology/s/revising-your-manuscript#loc-blurb>
Please below find our blurb:

When we change our mind in response to other people's opinion, we may be motivated to be correct or to have a good relationship with others. Using functional magnetic resonance imagining, we disentangle the neurobiological underpinnings of these two different motives in the human brain and show that the second motive is absent when we interact with computers.

7) Ethics: 

7.1) Please include the ID number of your protocol approved by the ethics committee at the University College London.

7.2) Please indicate if your study was conducted according to the principles expressed in the Declaration of Helsinki or any other specific national or international ethical guidelines.
We confirm that our study was conducted according to the Declaration of Helsinki. We have included this information, together with our ethics ID number in the methods.

8) Data: You may be aware of the PLOS Data Policy, which requires that all data be made available without restriction: <http://journals.plos.org/plosbiology/s/data-availability>. For more information, please also see this editorial: <http://dx.doi.org/10.1371/journal.pbio.1001797>

Note that we do not require all raw data. Rather, we ask for all individual quantitative observations that underlie the data summarized in the figures and results of your paper. For an example see here: <http://www.plosbiology.org/article/info%3Adoi%2F10.1371%2Fjournal.pbio.1001908#s5>

These data can be made available in one of the following forms:

8.I) Supplementary files (e.g., excel). Please ensure that all data files are uploaded as 'Supporting Information' and are invariably referred to (in the manuscript, figure legends, and the Description field when uploading your files) using the following format verbatim: S1 Data, S2 Data, etc. Multiple panels of a single or even several figures can be included as multiple sheets in one excel file that is saved using exactly the following convention: S1_Data.xlsx (using an underscore).

8.II) Deposition in a publicly available repository. Please also provide the accession code or a reviewer link so that we may view your data before publication. Please include a README file that explain how your data were analyzed to generate the plots of the figures mentioned below.

Regardless of the method selected, please ensure that you provide the individual numerical values that underlie the summary data displayed in the following figure panels: Figures 2B-E, 3, 4, 5AB, S1, S2, S3, S4, S5, S6, S7, S8, and S9.

NOTE: the numerical data provided should include all replicates AND the way in which the plotted mean and errors were derived (it should not present only the mean/average values).

8.1) Please also ensure that each figure legend in your manuscript includes information on where the underlying data can be found and that your supplemental data file/s has/have a legend.

8.2) Please ensure that your Data Statement in the submission system accurately describes where your data can be found.

We have uploaded all data and codes to create all figures in the manuscript and supplementary material to a public repository. The files can be found at <https://github.com/alimahmoodia/Reciprocity_Data/tree/main>
